# Arterial Lymphatics in Atherosclerosis: Old Questions, New Insights, and Remaining Challenges

**DOI:** 10.3390/jcm8040495

**Published:** 2019-04-11

**Authors:** Gábor Csányi, Bhupesh Singla

**Affiliations:** 1 Vascular Biology Center, 1460 Laney Walker Blvd., Medical College of Georgia, Augusta University, Augusta, GA 30912, USA; gcsanyi@augusta.edu; 2 Department of Pharmacology & Toxicology, 1460 Laney Walker Blvd., Medical College of Georgia, Augusta University, Augusta, GA 30912, USA

**Keywords:** lymphatic system, atherosclerosis, lymphangiogenesis, lymphatic vessels, reverse cholesterol transport, lymphatic endothelial cells

## Abstract

The lymphatic network is well known for its role in the maintenance of tissue fluid homeostasis, absorption of dietary lipids, trafficking of immune cells, and adaptive immunity. Aberrant lymphatic function has been linked to lymphedema and immune disorders for a long time. Discovery of lymphatic cell markers, novel insights into developmental and postnatal lymphangiogenesis, development of genetic mouse models, and the introduction of new imaging techniques have improved our understanding of lymphatic function in both health and disease, especially in the last decade. Previous studies linked the lymphatic vasculature to atherosclerosis through regulation of immune responses, reverse cholesterol transport, and inflammation. Despite extensive research, many aspects of the lymphatic circulation in atherosclerosis are still unknown and future studies are required to confirm that arterial lymphangiogenesis truly represents a therapeutic target in patients with cardiovascular disease. In this review article, we provide an overview of factors and mechanisms that regulate lymphangiogenesis, summarize recent findings on the role of lymphatics in macrophage reverse cholesterol transport, immune cell trafficking and pathogenesis of atherosclerosis, and present an overview of pharmacological and genetic strategies to modulate lymphatic vessel density in cardiovascular tissue.

## 1. Introduction

A network of lymphatic vessels (LVs), lymph nodes (LNs), and lymphoid organs, which play an important role in the maintenance of tissue homeostasis, constitute the lymphatic system [1]. The lymphatic network runs in parallel to the blood circulatory system and transports interstitial fluid, antigens, immune cells, inflammatory cytokines, and lipoproteins from the peripheral tissue to the surrounding lymph nodes and back to the systemic circulation, thereby playing major roles in parenchymal water homeostasis, host defense, adaptive immunity, and regulation of the inflammatory response [2,3,4]. The LVs are present in most of the internal organs and throughout the skin [5]. During embryogenesis, LVs originate from veins, and thereafter undergo extensive expansion by sprouting and proliferation, and form a hierarchical network of vessels categorized based on their specific functions and morphological features, namely lymphatic capillaries and collecting lymphatic vessels [6]. The flow of lymph through the lymphatic network is unidirectional and depends on extrinsic and intrinsic forces and one-way valves between the functional units of collecting lymphatic vessels, called lymphangions [7]. Impaired lymphatic function leads to pathological conditions, including inherited and acquired forms of lymphedema, malabsorption syndromes, autoimmune disorders, and immune deficiency [5,6,8].

Atherosclerosis is characterized by chronic inflammation and accumulation of lipids in the arterial wall [9]. Atherosclerosis and its cardiovascular consequences claim more lives than all types of cancer combined and it represents an enormous health care burden in western societies [10,11]. In recent years, the lymphatic network has attracted the attention of vascular biologists due to its role in immune cell trafficking, removal of cholesterol-loaded HDL from the periphery via reverse cholesterol transport (RCT), and the regulation of the inflammatory response in general [12,13,14,15,16]. Previous studies demonstrated the presence of LVs in atherosclerotic arteries, yet, our knowledge about the mechanisms regulating lymphangiogenesis and lymphatic function in the vessel wall and the role of LVs in the pathogenesis of atherosclerosis is limited. In this review article, we discuss the general anatomy and functions of LVs, provide an overview of factors and mechanisms that regulate lymphangiogenesis, highlight recent findings on the role of lymphatics in macrophage RCT and atherosclerosis, and present an overview of pharmacological and genetic strategies to regulate lymphangiogenesis in the vasculature.

## 2. Anatomy and Function of Lymphatic Vessels

The lymphatic vessels have a long history of recognition dating back to ancient Greece, where Hippocrates identified vessels containing “white blood” around 400 B.C. [6]. Lymphatic vessels were first described by Gaspare Aselli, an Italian physician, in 1627 as “lacteae venae” or milky veins, in the peritoneal cavity of a “well-fed” dog. Johann Vesling (1598–1649), a German anatomist and surgeon, produced the earliest illustrations of the human lymphatic system in his textbook in 1641. Subsequent studies in the 19th century demonstrated that the LVs constitute a network in mammals and are present in most of the tissues [17,18]. 

As mentioned above, LVs are classified into lymphatic capillaries and collecting LVs [6]. Lymphatic capillaries (also known as the initial lymphatics) are thin-walled (30–80 μm diameter) and blind-ended vessels that absorb lymph, a proteinous exudate from blood capillaries in the interstitial space, and drain into collecting LVs [19]. The lymphatic capillaries are composed of a single layer of lymphatic endothelial cells (LECs) that have discontinuous “button-like” junctions, which make them highly permeable [20]. Collecting LVs have a basement membrane with continuous “zipper-like” cell junctions and are covered by smooth muscle cells (SMCs). Active lymph transport by collecting LVs require exogenous forces, such as skeletal muscle contractions, arterial pulsation and inspiration, and endogenous forces provided by contraction of SMCs surrounding LECs. Contraction waves that are generated over the length of the lymphangions (contractile units) propel the lymph forward and the lymphatic valves located at the juncture of lymphangions prevent retrograde flow [14,21]. Collecting LVs drain lymph into chains of LNs and subsequently into the thoracic or right lymphatic duct, and the lymph ultimately reaches the low-pressure venous circulation through the right or left subclavian vein [17]. More details about the composition of lymph and its movement through the lymphatic network has been provided in previously published review article [22].

Previous studies identified various LEC-specific markers, including lymphatic vessel endothelial hyaluronan receptor 1 (LYVE-1) [23], podoplanin [24,25], prospero homeobox 1 (Prox1) [26], and vascular endothelial growth factor receptor 3 (VEGFR3) [27]. LECs present in lymphatic capillaries have a higher expression of LYVE-1 than that of collecting LVs [20]. Collecting LV LECs also express podoplanin, however, it is not expressed in LECs of lymphatic capillaries [21]. In humans, approximately 3 L of lymph is returned to the systemic circulation daily. The lymphatic system plays a key role in the removal of interstitial fluid from tissues, absorbs lipids from the gastrointestinal system, transports immune cells and pathogens from the periphery to LNs, and is a major site for dendritic cell-T-cell interaction and lymphocyte maturation. As such, a properly functioning lymphatic system is critical for the body to maintain fluid homeostasis, absorb fat and fat-soluble nutrients, and fight against infection.

## 3. Development of the Embryonic Lymphatic System

Florence Sabin was a medical researcher, best known for her work on blood cells and the lymphatic system. In 1902, she proposed that during embryonic development primary lymphatic sacs originate from the vascular endothelium of veins and other components of the lymphatic system are subsequently formed from LECs sprouting from these lymph sacs [17]. Later, it was discovered that during mouse embryonic day 9.5–10.5 (E9.5–E10.5), a subset of endothelial cells on the anterior cardinal vein starts expressing Prox1 under the regulation of transcription factor, SRY-related HMG-box 18 (Sox18) [28,29]. Prox1 is a transcription factor, which acts as a master regulator of lymphatic development [30]. In humans, Prox1 expression is induced during the 6th to 7th gestational week [8]. These Prox1-expressing endothelial cells subsequently become lymphatically committed and form lymph sacs and the lymphatic network throughout the body. The lymphatic system is absent in Prox1-knockout mice as sprouting, migration, and survival of these lymphatically-destined cells cease prematurely [31]. Subsequent studies identified other genes that play an important role in the development of mouse embryonic lymphatic system. Studies using VEGF-C-deficient mice revealed an indispensable role for the VEGF-C/VEGFR3 signaling pathway in early lymphatic development [32]. Activation of VEGFR3 by VEGF-C plays a pivotal role in the sprouting and migration of LECs and formation of lymph sacs. Interestingly, the structure and function of dermal LVs in VEGF-D-deficient mice appear to be normal and these mice do not develop any pathologic conditions consistent with a defect in lymphatic development [33]. Neuropilin-2, a co-receptor for VEGF-C, and ephrin B2, a ligand of Eph tyrosine kinase, are necessary for effective sprouting of lymphatics [34,35]. The hyaluronan receptor, LYVE-1 is expressed abundantly on LVs from early development and is one of the most widely used markers of LECs. Interestingly, mice deficient in LYVE-1 show no defect in lymphatic development and changes in secondary lymphoid tissue structure [36]. Contrary to these findings, Huang et al. demonstrated that LYVE-1-null mice have altered LV morphology in the liver and intestine, and PDGF-BB and HA enhance interstitial-lymphatic flow in wild type mice but not in knockout animals [37]. In addition to these signaling pathways, postnatal lymphangiogenesis has been shown to depend on Notch1-DII4 interaction [38]. Hence, the formation and maintenance of LVs depend on various mechanisms and a detailed description of these lymphangiogenic factors is given in Section 4. 

It is well established that Prox1-expressing venous endothelial cells transdifferentiate into LECs [39]. Interestingly, previous studies demonstrated the development of lymphatic vascular cells from nonvenous origin, leading to the concept of organ-specific development of lymphatic vasculature. Klotz et al. using multiple Cre–*lox*-based lineage tracing experiments demonstrated that yolk sac hemogenic endothelial cells (Tie2-lineage^−^ from nonvenous origin) are important sources of LECs in the mouse heart [40]. In addition, endothelial and non-endothelial cells involved in LN development have been observed to arise from nestin (a marker of mesenchymal stem cells) expressing precursor cells [41]. The skin, which is the first site of defense against infection, contains both LVs and blood vessels. Martinez-Corral et al. demonstrated that the majority of superficial dermal LVs in mice do not form from Tie2 positive venous endothelial cells. They reported that dorsal midline and lumbar region dermal LVs originate from non-Tie2-lineage cells via lymphvasculogenesis, however the exact identity of these precursor cells is still unclear [42]. In addition, LVs present in mesenteries develop from cKit lineage hemogenic endothelial cells [43]. Therefore, the origin of LECs present in different organs is diverse and more complex than we previously anticipated. The concept of organ-specific lymphatic vasculature has been recently reviewed by Petrova and Koh [44].

## 4. Molecular Factors Regulating Lymphangiogenesis

Like blood vessels, new LVs are directed by migrating tip LECs possessing cell membrane protrusions, which assist in sampling the microenvironment for guidance cues [45]. The stalk LECs behind tip cells undergo proliferation and ensure elongation of LVs. The elongation of LVs stops in the events of reduced concentration of growth factors (detailed below) or increased levels of endogenous anti-lymphangiogenic molecules, including IFN-γ, endostatin, TGF-β, neostatin-7, vasohibin, and semaphorins [46,47,48,49,50,51]. In adults, lymphangiogenesis is stimulated in pathological conditions, such as inflammation, tumor growth, and tissue repair [52]. These conditions are usually associated with tissue edema and accumulation of inflammatory cells, which necessitate lymphangiogenesis and remodeling of lymphatics for removal of tissue fluid, cytokines, chemokines, and immune cells [53]. In adults, lymphangiogenesis occurs similarly to embryonic lymphangiogenesis, however, it is less coordinated and dysregulated. VEGF-C does not promote significant lymphangiogenesis in adults unless notch signaling is suppressed [54]. Besides this, angiopoietins and their receptors (tyrosine kinase with immunoglobulin-like and EGF-like domains also known as Tie), are important for lymphangiogenesis [55]. Major molecular factors that regulate lymphangiogenesis during development and adults are described below:

### 4.1. VEGF-C/D and VEGFR3

VEGFR3 is highly expressed on lymphatic endothelial cells [56]. During the embryonic stage, VEGFR3 acts as a receptor for both VEGF-C and VEGF-D and its activation stimulates LV formation [32]. In adults, VEGFR3 expression is mainly limited to LECs and VEGF-C is indispensable for LV formation [27,37]. Hence, the VEGF-C/VEGFR3 signaling pathway is pivotal for both embryonic and adult lymphangiogenesis. Mature VEGF-C is formed by proteolytic cleavage of its precursor polypeptide [57]. The secreted mature disulfide-linked VEGF-C binds and activates VEGFR3 only, however, it can be further modified extracellularly by various proteases to form non-disulfide-linked VEGF-C, which can activate both VEGFR2 and VEGFR3 [58]. A positive correlation has been observed between the levels of hypoxia-inducible factor 1-alpha (HIF-1α) and VEGF-C-stimulated lymphangiogenesis in oral squamous cell carcinoma [59]. Interestingly, the promoter region of VEGF-C does not have any hypoxia response elements (HRE) [60], suggesting that HIF-1α cannot directly regulate VEGF-C transcription. Morfoisse et al. demonstrated the presence of internal ribosome entry site (IRES) on the 5′ UTR of VEGF-C mRNA and reported that hypoxia induces initiation of VEGF-C translation via an IRES-dependent mechanism and stimulates lymphangiogenesis [61]. Interestingly, a previous study by Milasan et al. demonstrated that treatment of LDLR^−/−^ mice with VEGF-C152S (a recombinant VEGFC analog that activates VEGFR3) attenuates atherosclerotic plaque development compared with control mice treated with control solution [62].

Heterozygous VEGF-C knockout (Vegfc^+/−^) mice have LV deficiency and develop lymphedema, while complete deletion of VEGF-C is embryonically lethal [32]. Likewise, inhibition of VEGFR3 signaling by overexpression of soluble VEGFR3 suppresses fetal lymphangiogenesis and induces regression of pre-existing lymphatics, without affecting the blood vasculature [63]. Collagen and calcium binding EGF domains 1 (CCBE1) augments the proteolytic activation of pro-VEGF-C to VEGF-C by ADAMTS3 (ADAM metallopeptidase with thrombospondin type 1 motif 3) metalloproteinase and promotes lymphangiogenesis [64,65]. Moreover, neuropilin-2, a transmembrane receptor for VEGF-C, interacts with VEGFR3 to enhance VEGFR3-mediated LV sprouting [35].

VEGF-D can activate both VEGFR3- and VEGFR2-mediated signaling in humans, however, it has been shown to stimulate only VEGFR3 in mice and induce lymphangiogenesis [66,67]. VEGF-D also stimulates angiogenesis [68]. Translation of VEGF-D is regulated by nucleolin-mediated IRES activation [69]. VEGF-D knockout adult mice have a slight decrease in bronchiolar lymphatics without any defect in lymphatic function [67]. These results suggest that VEGF-D is dispensable for lymphatic development or the presence of VEGF-C in these mice can compensate for the absence of VEGF-D during development. In contrast, VEGF-D overexpression has been shown to increase tumor lymphangiogenesis and metastasis [70,71]. Recently, the overexpression of VEGF-D specifically in the kidneys has been demonstrated to augment the density of renal lymphatics [72]. It has been shown utilizing a collar model of hyperplasia that adventitial VEGF-D adenoviral delivery promotes intimal hyperplasia in rabbits [73]. Furthermore, the expression of VEGF-C, VEGF-D, and LYVE-1 has been shown to be upregulated in human atherosclerotic arteries [74].

### 4.2. Prox1

Prox1 is a key transcription factor expressed in a subpopulation of endothelial cells that by budding and sprouting give rise to the lymphatic system during embryonic development [31]. Expression of Prox1 in cells destined to become LECs is regulated by Sox18 [28]. COUP transcription factor 2 (COUP-TFII) works in conjunction with Sox18 to promote Prox1 expression [75]. Prox1^+/−^ pups die by postnatal day 2 to 3 (P2–P3) due to fluid accumulation in the intestine, consistent with the role of Prox1 in the development of the lymphatic system in the gastrointestinal tract [31]. Prox1^−/−^ embryos do not survive beyond mid-gestational time (E14.5 to E15.0) due to developmental defects in the liver and lymphatic system. Importantly, vasculogenesis and angiogenesis are unaffected in Prox1-deficient mice, demonstrating that Prox1 signaling regulates primarily the lymphatic, but not the blood circulatory, system [31,76]. A previous study reported that activation of VEGFR3 signaling by VEGF-C contributes to increased Prox1 expression in LEC progenitors and differentiating LECs [77]. This study also demonstrated that Prox1 stimulates VEGFR3 expression in a dosage-dependent manner. The authors proposed that the Prox1-VEGFR3 feedback loop is an important sensing mechanism required to maintain the fate of LECs and regulate the number of specified LEC progenitors. Relevant to the pathogenesis of atherosclerosis, compromised interaction of SUMOylated liver receptor homolog 1 with prox1 has been demonstrated to downregulate hepatic expression of proteins involved in reverse cholesterol transport (ABCA1, ABCG1, and ABCG5) [78].

### 4.3. Podoplanin

Podoplanin is a widely used marker of fully differentiated LECs [24]. Uhrin et al. demonstrated that podoplanin plays an important role in the separation of lymphatic circulation from the blood vascular system during development [79]. Podoplanin expressed on developing lymphatic sacs interacts with circulating platelets from the cardinal vein via platelet C-type lectin-like receptor 2 (CLEC-2) and induces separation of lymphatic cells from blood vessels. High endothelial venules (HEVs) are found in secondary lymphoid organs and enable lymphocytes circulating in the blood to enter the lymphatic system. As demonstrated using inducible podoplanin null mice, podoplanin is also important for the integrity of HEVs and lymphocyte trafficking between the blood and lymphatic circulation [80]. Deletion of podoplanin is perinatally lethal and podoplanin knockout pups have congenital lymphedema, impaired lymphatic transport, undetectable lymphatic capillaries, and absence of abdominal lacteals [81]. A study by Hatakeyama et al. revealed increased expression of podoplanin in advanced atherosclerotic plaques compared to early lesions [82]. As podoplanin interacts with CLEC-2 present on platelets and can stimulate thrombus formation, it is possible that higher levels podoplanin contributes to thrombosis in advanced stages of atherosclerosis.

### 4.4. Sox18

Sox18 is a developmental transcription factor, expressed in LEC precursor cells and hair follicles [83]. It is required for inducing Prox-1 expression in lymphatically committed cells and Sox18-deficient embryos have a complete blockade of LECs differentiation from cardinal vein endothelial cells [28]. In humans, Sox18 mutations have been reported to cause hypotrichosis, lymphoedema, and telangiectasia (a.k.a. HLT syndrome), suggesting its crucial role in the development and maintenance of the lymphatic system [84]. García-Ramírez et al. investigated the expression of SOX18 in human coronary atherosclerotic lesions and its function in vascular cells [85]. Immunostaining of SOX18 in endothelial and vascular SMC (VSMC) revealed its co-localization with a key DNA replication factor, proliferating cell nuclear antigen. Depletion of SOX18 reduces endothelial, as well as VSMC proliferation, suggesting its role in arterial remodeling.

### 4.5. LYVE-1

LYVE-1 is a CD44 homolog and a major receptor of hyaluronan (HA), specifically present on the LEC surface [23]. It is involved in HA-induced lymphangiogenesis via activation of intracellular protein kinase C α/βII and ERK1/2 [86]. Wong et al. demonstrated that membrane type-1-matrix metalloproteinase cleaves LYVE-1 on LECs and inhibits LYVE-1-mediated lymphangiogenic responses [87]. Interestingly, mice deficient in LYVE-1 show no defect in lymphatic development and changes in secondary lymphoid tissue structure, probably because of the functional compensation by CD44 [36]. Contrary to these findings, Huang et al. demonstrated that LYVE-1-null mice have altered LV morphology in the liver and intestine, and PDGF-BB and HA enhance interstitial-lymphatic flow in wild type controls but not in knockout animals [37]. LYVE-1 is also expressed in a subset of infiltrating macrophages. Recently, Lim et al. reported that LYVE-1^+^ macrophages associate with vascular smooth muscle cells, and specific deletion of aortic-resident LYVE-1^+^ macrophages leads to arterial stiffness due to reduced MMP-9 levels [88]. 

### 4.6. Angiopoietin 2

The angiopoietins (Ang) are a family of secreted vascular growth factors comprising Ang1, Ang2, and Ang3 (Ang4 in humans). These angiopoietins and their receptors, Tie1 and Tie2, are involved in the regulation of lymphangiogenesis. Previous studies demonstrated that Ang1 overexpression stimulates lymphatic sprouting and growth via promoting Tie2-mediated signaling [89,90]. In addition, murine Ang3 and human Ang4 also induce LV formation [91]. Ang2 plays an important role in lymphatic development during both the embryonic and neonatal phases. Deletion of Ang2 in mice leads to severe defects in the maturation of collecting LVs, and dermal LVs fail to undergo postnatal remodeling in Ang2^−/−^ mice [92,93]. Most of Ang2^−/−^ mice do not survive more than 2 weeks after birth and suffer from chylous ascites and peripheral edema. These studies suggest the indispensable role of Ang2 in normal lymphatic function and development. Ang2 works as an agonist for LV development and as an antagonist for vascular blood vessel formation [92,94]. Deletion of Tie1 receptor also prevents the proper development of lymphatic vasculature [95]. 

Ang2 has been shown to stimulate leukocyte recruitment, angiogenesis and vascular permeability [96]. Therefore, increased Ang2 expression may contribute to the development of atherosclerosis independent of its effect on lymphangiogenesis. Theelen et al. demonstrated that treatment with an anti-Ang2 antibody reduces the size of fatty streaks in the brachiocephalic artery of LDLR^−/−^ apoB^100/100^ mice [97]. Furthermore, upregulated Ang2 expression in human atherosclerotic carotid arteries has been observed to correlate with increased metalloproteinases activity and plaque rupture [98]. On the contrary, adenoviral-mediated overexpression of Ang2 in ApoE^−/−^ mice reduced the size of atherosclerotic lesions and inhibited LDL oxidation via NO-dependent pathways [99].

### 4.7. EphrinB2

EphrinB2, a transmembrane ligand, and its cognate receptor, EphB4, are important for embryonic blood vascular morphogenesis [100]. EphrinB2 is highly expressed in the valves of collecting LVs, and involved in the regulation of lymphatic valve formation and sprouting of lymphatic capillaries [5,34]. A previous study demonstrated an association between VEGF-C/VEGFR3 and EphB4/ephrinB2 signaling mechanisms. Stimulation of EphB4-ephrin signaling induces VEGFR3 internalization into endosomes in LECs [101]. In ephrinB2-null LECs, VEGFR3 internalization is impaired, which compromised downstream VEGFR3-mediated growth signaling as full VEGFR3 signaling is coupled to receptor internalization [101]. Recently, it has been shown that selective inhibition of EphB4 using a blocking antibody results in defective lymphatic valve development [102]. 

### 4.8. Forkhead Box C2

Forkhead Box C2 (FoxC2) is a transcription factor that is essential for correct lymphatic remodeling, lymphatic valve formation and maturation of collecting LVs [103]. FoxC2 point mutations in humans cause a rare genetic multisystem disorder called lymphedema-distichiasis syndrome, which is characterized by the development of extra eyelashes, functionally defective lymphatic valves and presence of lymphedema [104,105]. FoxC2 is required for the maintenance of postnatal collecting LVs in oscillatory shear stress conditions due to its role in stabilization of LEC intercellular junctions and cytoskeleton [106]. Similar to humans, FoxC2 haploinsufficient mice exhibit lymph node hyperplasia and lymphedema of hind limbs and display distichiasis [107]. Recently, Fatima et al. have reported that mice lacking FoxC1 and 2 have aberrant expression of Ras regulators and exhibit ERK hyperactivation [108]. In this study, pharmacological ERK inhibition in utero abolished the abnormally enlarged LVs in FOXC-deficient embryos, suggesting the role of Ras/ERK signaling pathway in FoxC1/C2-regulation of LV development. 

### 4.9. Neuropilin 2

Neuropilin 2 (Nrp2) acts as a co-receptor for VEGF-C and promotes lymphangiogenesis [109]. During early embryonic stages (E10), Nrp2 is expressed in veins, but not in the lymphatic endothelium. Starting from embryonic day E13, Nrp2 starts expressing in lymphatic vessels [110]. Nrp2 promotes lymphangiogenesis via inducing sprouting of lymphatics from pre-existing LVs [35,110]. Homozygous Nrp2 mutant mice have markedly decreased numbers of lymphatic capillaries, however, these mice have a normal development of collecting LVs, indicating the selective requirement of Nrp2 for the formation of small lymphatic capillaries [110]. Caunt et al. have demonstrated that an antibody blockade of Nrp2 disrupts VEGF-C-induced migration of LECs, inhibits tumor lymphangiogenesis, and reduces metastasis formation [111]. 

### 4.10. Collagen and Calcium-Binding EGF Domain-Containing Protein 1

Hogan et al. identified collagen and calcium-binding EGF domain-1 (CCBE1) as a crucial player for embryonic lymphangiogenesis in zebrafish using a genetic screen [112]. In this study, it has been shown that CCBE1 is necessary for budding, migration, and proliferation of lymphangioblasts. Mutations in CCBE1 may cause primary generalized lymph vessel dysplasia in humans. Moreover, mutations in CCBE1 were identified in a subset (23%) of patients diagnosed with Hennekam syndrome, an inherited disorder resulting from malformation of the lymphatic system [113]. Subsequent studies confirmed the role of CCBE1 in lymphangiogenesis using murine models [114]. CCBE1 has little lymphangiogenic activity on its own but it significantly upregulates VEGF-C-mediated lymphangiogenesis in vivo [114].

### 4.11. Sphingosine-1-Phosphate

Sphingosine-1-phosphate (S1P) is a bioactive lipid mediator, which is involved in multiple important physiological processes, including cell survival and growth, angiogenesis, regulation of vascular tone, and immunity [115]. S1P is also a well-known regulator of blood vessel formation [116,117]. S1P promotes lymphangiogenesis in vitro via activation of S1P1/Gi/PLC/Ca^2+^ signaling [118]. It has been demonstrated that S1P induces exocytosis of Ang2 from LECs, which is required for lymphatic development [119]. Deletion of SPHK1 (a kinase required for phosphorylation of sphingosine) from LECs in mice suppresses maturation of LVs [120]. S1P mediates ABCA1-mediated cholesterol efflux from macrophages, therefore may play a role in macrophage RCT [121].

### 4.12. Hepatocyte Growth Factor

Hepatocyte growth factor (HGF) belongs to the plasminogen-prothrombin gene superfamily, although, it does not possess any proteolytic activity unlike other members in the family [122]. It needs to be proteolytically cleaved by serine proteinases to promote cellular growth [123]. HGF has been reported to stimulate lymphangiogenesis via indirectly activating VEGFR-3 signaling [124]. In addition, HGF is also a potent angiogenic factor [125]. Overexpression of HGF via plasmid transfer promotes lymphangiogenesis and attenuates accumulation of interstitial fluid in a rat tail lymphedema model [126].

## 5. The Role of the Lymphatic System in Atherosclerosis

### 5.1. Atherosclerosis

Atherosclerotic cardiovascular disease accounts for one out of every three deaths in the United States and it is one of the most serious health problems of the western world [127]. Atherosclerosis is initiated by subendothelial retention of apolipoprotein B (apoB)–containing lipoproteins in focal areas of arteries, particularly regions in which laminar flow is disturbed by bends or branch points in the arteries [128]. Various modifications of the retained lipoproteins likely mimic pathogen- and damage-associated molecular patterns and thereby trigger a low-grade inflammatory response. Circulating cardiovascular risk factors and the low-grade arterial inflammation leads to activation of endothelial cells and the development of endothelial dysfunction. In response, endothelial cells express adhesion molecules, which mediate adhesion of circulating monocytes to the endothelium and their subsequent infiltration into the arterial wall [129]. The transmigrated monocytes differentiate into macrophages and internalize modified lipoproteins to form lipid-laden foam cells. Arterial smooth muscle cells also internalize lipids and contribute to the pathogenesis of atherosclerosis [130,131]. The arterial lipid deposits and inflammatory response promote cell proliferation within the arterial wall and stimulate tissue remodeling that may gradually impinge on the vessel lumen and impede blood flow. This process often lasts for decades until an atherosclerotic lesion, through arterial remodeling and hemodynamic forces from blood flow, becomes unstable and ruptures [132]. As a consequence of rupture, deep arterial wall components are exposed to blood flow, leading to platelet activation, thrombosis, and compromised oxygen supply to vital organs, such as the heart and brain [133]. Ischemic coronary artery disease (CAD) and stroke are the predominant causes of death and morbidity worldwide [134]. 

**Figure 1:**
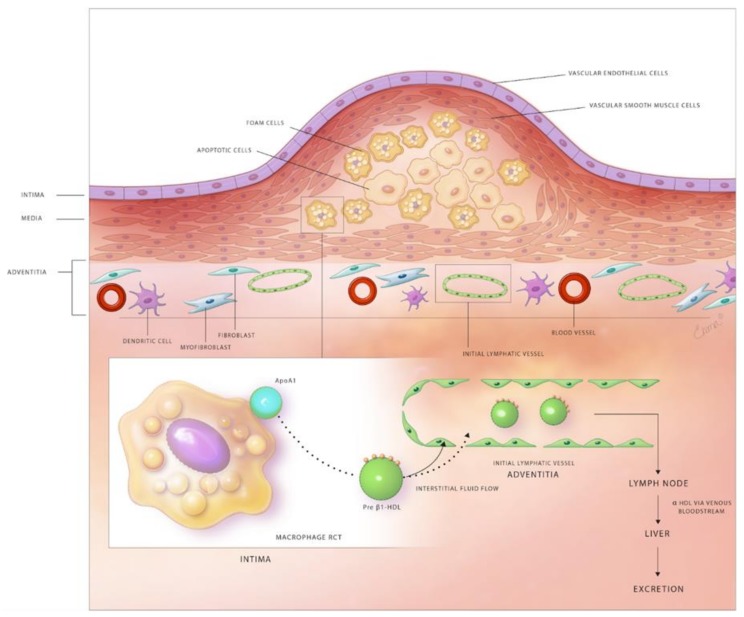
Schematic diagram depicting the role of lymphatic vessels in reverse cholesterol transport in the atherosclerotic arterial wall. Subendothelial lipid-laden foam cells efflux free cholesterol through the plasma membrane transporters, ABCA1 and ABCG1. The exported free cholesterol is taken up by the apoA1 component of HDL to form pre-β1 HDL. Pre-β1 HDL moves from atherosclerotic lesions to the adventitial area, where lymphatics exist via the flow of interstitial fluid. Pre-β1 HDL lipoproteins enter the lymphatic circulation either by SR-B1-mediated transcytosis across LECs of initial lymphatics or via paracellular transport between LECs. Semilunar lymphatic valves between lymphangions, endogenous forces provided by the contraction of lymphatic SMCs, and exogenous forces, including skeletal muscle contractions, arterial pulsation, and inspiration maintain the unidirectional flow of lymph through the lymphatic network. Pre-β1 HDL finally reaches the low-pressure venous circulation via the subclavian vein(s), becomes modified to α-HDL, which is then internalized by hepatic cells and excreted into the gastrointestinal system.

Experimental and clinical studies suggest that at least partial regression of atherosclerotic lesions can be achieved by therapeutic and genetic interventions [135]. Possible mechanisms responsible for plaque regression include decreased retention of LDL within the arterial wall, stimulated efflux of cholesterol loaded onto HDL from plaques, emigration of foam cells out of the arterial wall, and the influx of healthy phagocytes that remove apoptotic cells and necrotic debris from atherosclerotic lesions. The lymphatic network and macrophage RCT are important targets to attenuate lesion development and stimulate plaque regression (Figure 1).

### 5.2. Lymphatic Vessels in Atherosclerotic Arteries

The presence of lymphatics in the arterial wall was described by Hoggan et al. more than one hundred years ago [136]. The anatomy of lymphatics of blood vessels in dogs, pigs, and humans was described by subsequent studies in the 20th century [137,138]. Drozdz et al. discovered LVs in the adventitial regions of human atherosclerotic carotid arteries and demonstrated that LV number increases with the severity of atherosclerotic disease. The authors proposed that the inflammatory environment promotes lymphangiogenesis in atherosclerotic vessels [139]. The presence of LVs in the adventitial layer of atherosclerotic arteries was confirmed by other investigators [140]. Contrary to these findings, Eliska et al. and Nakano et al. detected no or very low number of LVs (compared to blood vessels) in normal and atherosclerotic human coronary arteries despite high levels of VEGF-C in the arterial wall [141,142]. Recently, Kutkut et al. have demonstrated the existence of LVs in adventitial as well as intraplaque regions of human carotid endarterectomy specimens [143]. Nearly 30 years ago, Miller et al. proposed that disruption of cardiac lymphatic drainage in allogenic transplanted hearts may be the cause of accelerated atherosclerosis in coronary arteries [144]. Contrary to this hypothesis, Xu et al. proposed that adventitial lymphatics play an important role in the development of atherosclerosis by enhancing the activation of inflammatory cells and stimulating inflammation in the arterial wall [145]. Despite the documented presence of lymphatic vessels in the arterial wall and conflicting theories regarding their role in atherosclerosis, the functional role of lymphatics in the pathogenesis of atherosclerosis has not been investigated until recently.

### 5.3. Macrophage Reverse Cholesterol Transport (RCT), HDL and Clinical Trials

Macrophage RCT is a term used to describe the efflux of cholesterol from macrophages localized in peripheral tissues, including atherosclerotic vessels, and its excretion via liver to the bile and ultimately to the feces [146,147]. Cholesterol is effluxed from macrophages to extracellular HDL through the action of plasma membrane transporters, including ATP binding cassette transporter A1 (ABCA1) and ATP-binding cassette transporter G1 (ABCG1) [148]. It has been shown that myeloid cell-specific deletion of ABCA1 and ABCG1 aggravate atherosclerotic lesion formation [149,150]. A previous study by Trigueros-Motos et al. sequenced ABCA8 gene of Dutch Caucasian subjects with low and high plasma HDL levels [151]. They detected three deleterious ABCA8 mutations (P609R, E760, and T741X) in individuals with low HDL levels. The authors also observed a significant increase in plasma HDL with ABCA8 overexpression in mice and increased reverse cholesterol transport to the liver. These results suggest that ABCA8 plays an important role in HDL metabolism in both humans and mice. The HDL particles can leave the arterial wall as a component of interstitial fluid through the lymphatic system. The liminal arterial endothelial cells are anatomically closer to lipid-laden macrophages in atherosclerotic arteries. Relevant to this point, it is possible that HDL particles can leave atherosclerotic arteries through the luminal endothelial layer, although, there is no direct evidence to support this statement. The precise mechanisms that determine the “fate” of cholesterol-loaded HDL in atherosclerotic lesions and the efficiency of RCT are not well understood. The transport of HDL through the lymphatic system has been expertly reviewed by Randolph and Miller [4]. 

In the past decade, multiple clinical trials have aimed to decrease cardiovascular morbidity and mortality in patients with established CAD by increasing plasma HDL levels and stimulating macrophage RCT. Unfortunately, these trials reported that HDL-mimetic agents do not improve RCT, reduce atherosclerotic lesions or improve clinical outcomes in CAD patients [152,153]. These unexpected outcomes can be explained by the fact that not all HDL particles are equally capable of stimulating macrophage cholesterol efflux and the cholesterol-loaded HDL particles can be still “trapped” in lesions and unable to return to the systemic circulation (and liver) with their lipid cargo [154].

### 5.4. Role of Lymphatics in Macrophage RCT and Pathogenesis of Atherosclerosis 

Previous studies utilized genetic, surgical and pharmacological tools to disrupt lymphatic drainage in animal models in order to better understand the relationship between the lymphatic network and RCT [13,15,16]. Martel et al. transplanted the aorta of ApoE-deficient mice loaded with [^2^H]_6_-labeled cholesterol to ApoE null recipients treated with a VEGFR3-blocking or control antibody and ApoE adenoviral vector [13]. In these experiments, the recipient mice were treated with an anti-VEGFR3 antibody to prevent the development of lymphatic connections between the transplanted aorta and surrounding tissue. The authors demonstrated that [^2^H]_6_-labeled-cholesterol was retained in the aorta of anti-VEGFR3, but not in control antibody-treated mice, suggesting that the lymphatic network plays a critical role in macrophage RCT from the arterial wall of atherosclerotic mice. To our knowledge, this is the only study to date that has evaluated RCT from atherosclerotic arteries. Consistent with this observation, genetic ablation of lymphatic vessels in Chy mice, haploinsufficient VEGFC mutants with hypoplastic dermal lymphatic network, inhibits RCT from the skin [13]. It is important to note that arterial surgical interventions used in this study to investigate RCT promote inflammation and LV development, such that the cholesterol drainage is inflammation-dependent even in control mice without blocking antibody treatment [155]. Thus, further investigations are important to determine the contribution of LVs in RCT using nonsurgical models. Vuorio et al. utilized two models of lymphatic insufficiency [Chy mice and soluble vascular endothelial growth factor 3 (sVEGFR3)-overexpressing mice] and crossed them with atheroprone mice (LDLR^−/−^/ApoB^100/100^) to investigate the effects of impaired lymphatic transport on lipoprotein metabolism [16]. They observed significantly elevated plasma cholesterol levels in both lymphatic insufficiency models compared to controls with both normal chow and western diet feeding. However, they did not find a difference in in vivo RCT after intraperitoneal injection of radioactively labeled-macrophages between sVEGFR3×LDLR^−/−^/ApoB^100/100^ mice and LDLR^−/−^/ApoB^100/100^ controls. The possible reasons for similar RCT in these mice include unaltered peritoneal lymphatics by sVEGFR3-overexpression and impaired lymphatic function in control mice due to hypercholesterolemia [12]. The authors postulated that arterial wall adventitial lymphatics might aid in mobilization of unretained cholesterol and lipoproteins out of the vessel wall in addition to macrophage RCT. Hypercholesterolemic ApoE knockout mice have been shown to have decreased expression of lymphangiogenic factors, including VEGF-C, Ang2, and FoxC2 in peripheral tissues and insufficient lymphatic drainage [12]. The restoration of lymphatic function via VEGF-C treatment in these hypercholesterolemic mice improves cholesterol clearance from the peritoneal cavity and skin. Furthermore, Lim et al. discovered that removal of cholesterol by lymphatic vessels is dependent on the transcytosis of HDL by scavenger receptor class B type I (SR-B1) expressed on LECs [12] (Figure 1).

Though VEGF-C levels are upregulated in the atherosclerotic vessel wall, adventitial lymphatics undergo regression with the progression of atherosclerotic disease. Taher et al. reported upregulated expression sVEGFR2 in the aorta of aged and western diet-fed atherosclerotic ApoE null mice, which binds to VEGF-C and impede maintenance of lymphatic vasculature in the aortic wall [156]. This lymphatic regression may contribute to exacerbated atherosclerosis. It has been shown that apoA-I treatment improves lymphatic transport, abolishes collecting LV permeability, and reduces aortic lipid accumulation without affecting total cholesterol levels by strengthening junctions between LECs [157]. However, no prior studies have tested whether stimulation of lymphangiogenesis or lymphatic function increases reverse cholesterol transport from atherosclerotic arteries. The detailed role of lymphatics in cholesterol transport has been expertly reviewed previously [158].

Despite the important role of lymphatics in RCT and immune cell trafficking, only a few studies have investigated the direct association between lymphatic transport and atherosclerotic lesion formation. As described above, Vuorio et al. employed two murine models of impaired lymphatic vessel function on atherosclerotic background to study the effects of lymphatic insufficiency on the development of atherosclerosis (see Table 1) [16]. The authors detected increased plasma cholesterol levels in both models of lymphatic insufficiency compared with controls, however, accelerated atherosclerotic lesion formation was only occurred in atheroprone soluble VEGFR3 (sVEGFR3)-overexpressing mice compared to control mice. Milasan et al. characterized the development of lymphatic vessel dysfunction along the progression of atherosclerosis [14]. The authors showed improved collecting LV function and elevated LDLR expression on LECs in atheroprotected PCSK9-deficient mice (PCSK9 causes lysosomal degradation of low-density lipoprotein receptor, LDLR). LDLR^−/−^; hApoB^100/100^ mice are severely dyslipidemic and develop atherosclerosis at the age of 4 months on regular chow diet. It has been demonstrated that these mice have a dysfunctional lymphatic network before the onset of atherosclerotic lesion formation at the age of 3 months [14]. Treatment of atheroprone, LDLR^−/−^; hApoB^100/100^ mice with VEGF-C152S, a VEGFR-3 agonist, prevents lymphatic function impairment, advocating the correlation between LDLR modulation and lymphatic function [14]. Another study from the same research group has reported improved lymphatic transport and reduced permeability of collecting LVs in LDLR^−/−^ mice treated with lipid-free apoA-I compared to control mice [157]. The authors observed plaque regression in the thoracic aorta of apoA-I-treated mice independent of plasma and lymph cholesterol accumulation. The unchanged plasma cholesterol levels may be because of more potent efficacy of apoA-I in cholesterol mobilization from immune cells than regulating whole-body cholesterol equilibrium [159]. A recent study by Milasan et al. treated LDLR^−/−^ mice systemically with VEGF-C152S and control solution before the onset of atherosclerosis and reported decreased plaque development in VEGF-C152S-treated mice compared with controls [62]. They concluded that early VEGF-C treatment increased contraction frequency of the collecting LVs and lymphatic transport, leading to attenuated development of atherosclerosis in Western diet-fed mice. In previous studies performed by Milasan et al. [14,62,157], the authors’ determined function and permeability of dermal lymphatics including popliteal LVs, back skin, and ear LVs. Importantly, the above studies did not investigate arterial LV function which would be more relevant to atherosclerosis development as the lymphatic function can vary in different organs and tissues. In addition, most of the previous studies did not provide 3D visualization of lymphatics or investigated the pumping rate of lymphangions so information about the overall flow rate through arterial wall lymphatics is still missing. It is also possible that the overall lymphatic drainage is still increased in inflammatory conditions, even though lymphatics are leaky. Previous studies have demonstrated the feasibility of quantitation of overall lymph flow in real time in hindlimb, ear and back skin [160,161,162]. These methods would be useful in the assessment of total lymph flow in atherosclerotic models. Rademakers et al. showed enhanced atherosclerotic lesion formation in the carotid artery following surgical disruption of periarterial lymph flow in ApoE knockout mice [15]. A recent study by Tirronen et al. studied the effects of VEGF-D (a lymphangiogenic factor) deletion on lipid metabolism in atherogenic LDLR^−/−^ApoB^100/100^ mice [163]. They observed significantly elevated levels of cholesterol and triglyceride in plasma of VEGF-D^−/−^LDLR^−/−^ApoB^100/100^ mice compared with LDLR^−/−^ApoB^100/100^ mice. This was due to decreased expression of hepatic syndecan 1 in VEGF-D-deficient mice, which is involved in chylomicron remnant uptake by hepatocytes. However, they did not observe a difference in atherosclerotic plaque area between groups and investigate lymphatic density and function. Taken together, these previous studies hint towards the possible association between lymphatic function and atherosclerosis, however, more future studies are required to strengthen this link. 

### 5.5. Trafficking of Cytokines and Immune Cells through the Lymphatic Network

Lymphatics are crucial conduits in draining cytokines from the interstitial fluid at the site of inflammation. In addition, LVs play a pivotal role in the initiation of immune responses via providing a route for antigen presenting cells from peripheral infection sites to draining LNs. Lymphatics also play an important role in the removal of leukocytes and cytokines from atherosclerotic plaques [164]. Interestingly, reduced egression of immune cells from the aortic wall was observed during the progressive stage of atherosclerotic lesion development in ApoE-deficient mice [164,165]. Llodrá et al. utilized aortic transplantation to study cell egress from atherosclerotic lesions [164]. There are limitations to this approach that need to be appreciated. Firstly, the authors have used an aortic transplantation model, an approach that induces inflammation in the periarterial region where draining LNs reside and aortic tissue; secondly, they did not provide any evidence of mobilization of these cells through the medial layer to reach the adventitial lymphatics. Till date, it is not known how lipid-laden macrophages present in the subendothelial layer of atherosclerotic arteries migrate through the thick medial layer, and from there drain into the lymphatics to reach LNs and systemic circulation. A recent study from the same research group reported that macrophages present in plaques (progressing and regressing) do not egress, supporting the idea that the migration through the medial layer may not occur [166]. Mueller et al. using an intravenous injection of EdU-labelled CD11b^+^ monocytes reported the emigration of these cells into mediastinal LNs from plaque, however, they did not show any evidence of recruitment of these cells to the atherosclerotic lesion, and their egress from lesion [167]. Recently, Rademakers and his coworkers showed that dissection of carotid artery draining LNs and LVs in ApoE^−/−^ mice enhances atherosclerotic lesion formation due to increased accumulation of intimal and adventitial CD3^+^ T cells [15]. In addition, inhibition of VEGFR3-mediated signaling caused T-cell enrichment in atherosclerotic lesions, without compromising adventitial lymphatic density, suggesting the involvement of VEGFR3-independent growth of lymphatics. The authors identified the CXCL12/CXCR4 signaling pathway as an alternate mechanism responsible for lymphangiogenesis in the event of impaired VEGFR3-mediated signaling [15]. However, the authors did not investigate migration of cells out of plaques into lymphatics; plus, the semi-constrictive collar placed on carotid arteries may compress adventitial lymphatics, which could have unknown effects.

The entry and transport of immune cells through lymphatic vessels are controlled by LECs via expression of various adhesion molecules and chemokines. LECs express CCL21 and CCL19, which guide and help in homing of CCR7 positive mature DCs, macrophages and other immune cells to lymphatic capillaries and LNs [168,169,170]. Milasan et al. investigated DC mobilization through lymphatics to draining lymph nodes, however, the authors did not examine the levels of CCL21 and CCL19, and CCR7 receptor expression on the surface of DCs [14,62,157]. It is important to consider the expression of these chemokines and receptors while evaluating lymphatic function. Karlsen et al. reported that transgenic mice overexpressing VEGF-C in keratinocytes have an expanded lymphatic network in the skin, leading to enhanced initial lymph formation compared with control mice [171]. The increased lymph vessel area resulted in enhanced production of CCL21 that, however, did not lead to augmented DC migration to LVs after stimulation with fluorescein isothiocyanate. The explanation might be the reduced CCR7 expression in DCs in VEGF-C overexpressing mice [172]. Importantly, these results demonstrate that lymphatic trafficking of immune cells depends on mechanisms that are independent of the density of initial lymphatics, which warrants the need for future mechanistic studies. Overexpression of VEGF-D specifically in the kidney has been shown to induce renal lymphatic vessel formation and lead to reduced renal accumulation of macrophages and T-cells compared with control mice [72]. A recent study by Huang et al. utilized a novel photoactivatable apoA-I to study endogenous apoA1 HDL trafficking in mice with psoriasis (a skin condition that increases risk of cardiovascular diseases) and demonstrated reduced HDL trafficking due to increased thickening of the collagenous matrix around the carotid artery [173]. They also observed increased atherosclerosis in psoriatic ApoE^−/−^ mice compared with controls, which was inhibited by anti-IL17 neutralizing antibody and lysyl oxidase inhibitor treatment. The migration of DCs to lymph nodes, lymph flow (as measured by intravital 2-photon microscopy), and albumin transit from the skin to plasma were normal, suggesting that decreased HDL trafficking is not always related to lymphatic drainage and it may depend on the size of molecules and other properties [173]. A better understanding of the factors and mechanisms that regulate the migration of immune cells from the site of inflammation to regions where lymphatic capillaries are present is required.

## 6. Conclusions and Future Perspectives

Several studies have evaluated the therapeutic potential of stimulating lymphangiogenesis via treatment with recombinant VEGF-C/D or adenovirus-mediated overexpression of VEGF-C/D in various pathologies, including atherosclerosis, inflammatory bowel disease, myocardial infarction, and hind limb ischemia, and reported promising results [12,14,174,175,176,177]. Earlier studies have demonstrated the possibility of differentiating human endothelial precursor cells into LECs in vitro [178]. The efficacy of endothelial precursor cell transplantation, however, to stimulate lymphangiogenesis in the arterial wall in vivo has not been studied. Intramyocardial injection of EphB4 has also been shown to promote lymphatic regeneration and improve cardiac function in mice after myocardial infarction [179], suggesting EphB4 as a potential therapeutic molecule to stimulate lymphatic vessel formation in the heart. The knowledge gained so far about regulating mechanisms of lymphangiogenesis in atherosclerotic vessels is from animal studies, including mainly rodents, which possesses a challenge of translating the obtained results to humans. Nonetheless, the induction of lymphangiogenesis may promote metastasis, and therefore the safety of lymphangiogenic therapy should be carefully tested in the context of each pathological condition. Inhibition of anti-lymphangiogenic signaling may be also an innovative approach to stimulate lymphatic drainage in atherosclerotic arteries and induce plaque regression. 

The levels of HDL is significantly higher in the lymph compared to blood, advocating an important role of lymphatics in RCT [180]. However, the majority of previous studies investigated lymphatic function either in dermal tissue or peritoneal cavity and correlated it with RCT and development of atherosclerosis. The lymphatic drainage varies among tissues. Therefore, future studies are needed to determine RCT from atherosclerotic arteries via lymphatics and assess its effect on atherosclerosis. Furthermore, methodological techniques to investigate overall lymph flow rate should be employed to determine lymphatic drainage. One should also keep in mind that cholesterol efflux from macrophages is the rate-limiting step in RCT and therefore stimulating lymphangiogenesis per se may not induce plaque regression. It is possible that combinatorial therapies stimulating cholesterol efflux from macrophages, increasing HDL levels and induction of arterial lymphangiogenesis are all needed to stimulate RCT and induce plaque regression. Clearly, more studies are required to better understand the role of lymphatics in the pathogenesis of atherosclerosis and whether stimulation of lymphangiogenesis truly represents a therapeutic target in patients with atherosclerotic vascular disease.

## Figures and Tables

**Table 1:** Role of lymphatics in mRCT and immune cells trafficking in the arterial wall.

Reference	Murine Model	Intervention/Treatment	Site of Lymphatic Function Assessment	Outcome
Martel et al. [13]	WT C57BL/6	Disruption of tail lymphatics and injection of [^3^H]-cholesterol–loaded macrophages into the tail distal to lymphatic disruption	Tail	Reduced [^3^H]-Cholesterol levels in plasma, liver and feces of mice with lymphatic disruption
Martel et al. [13]	Chy mice	Injection of [^3^H]-cholesterol–loaded macrophages into the footpads	Footpad skin	Decreased RCT in Chy mice compared with control mice
Martel et al. [13]	ApoE^−/−^ Regression model	Transplantation of [^2^H]_6_−labeled cholesterol-loaded aorta to ApoE^−/−^ mice treated with a VEGFR3 blocking antibody ApoE adenoviral vector	Aorta	Reduced RCT in VEGFR3 blocking antibody-treated mice
Vuorio et al. [16]	Chy×LDLR^−/−^/ApoB^100/100^ sVEGFR3×LDLR^−/−^/ApoB^100/100^	Normal chow diet or Western diet		Elevated plasma cholesterol levels in Chy mice compared with control mice on chow as well as Western diet No change in plasma cholesterol levels among sVEGFR3×LDLR^−/−^/ApoB^100/100^ mice and respective controls Accelerated atherosclerosis in young and middle-aged sVEGFR3 mice compared with control mice after 6 weeks of Western diet
Lim et al. [12]	ApoE^−/−^	Regular chow diet and VEGF-C treatment Surgical disruption of afferent lymphatic vessels	Peritoneal cavity and skin	Improved lymphatic transport and reduced peripheral lipid accumulation in VEGF-C-treated mice. Decreased RCT in mice with excised lymphatic vessels
Milasan et al. [14]	Pcsk9^−/−^ LDLR^−/−^; hApoB^100/100^	Regular chow diet and VEGF-C152S treatment	Footpad and back skin	Improved collecting lymphatic vessel function in Pcsk9^−/−^ mice. VEGF-C-treated LDLR^−/−^; hApoB^100/100^ mice
Taher et al. [156]	ApoE^−/−^	Western diet		Decreased lymphatic vessel density in aged and Western diet-fed mice due to increased expression of sVEGFR2 in aortic tissue
Milasan et al. [157]	LDLR^−/−^	Western diet and apoA-I treatment	Footpad and back skin	Enhanced lymphatic transport, improved LV permeability and reduced atherosclerotic lesion formation in apoA-I-treated mice
Rademakers et al. [15]	ApoE^−/−^	Western diet Semi-constrictive collars on carotid artery Dissection of plaque draining lymph node and lymphatic vessel		Aggravated atherosclerotic lesion formation Accumulation of CD3^+^ T cells in the carotid artery
Rademakers et al. [15]	ApoE^−/−^	Western diet AAV-hVEGFR3 treatment		Accumulation of CD3^+^ T cells in the intima and adventitia
Milasan et al. [62]	LDLR^−/−^Regression model	VEGF-C152S pretreatment Western diet for 8 weeks and one group switched to regular chow diet after 8 weeks	Footpad and back skin	VEGF-C pretreatment improved lymphatic transport and attenuated plaque formation in mice sacrificed after Western diet period
